# Comment on ‘Carbon and fullerene nanomaterials in plant system’

**DOI:** 10.1186/s12951-016-0180-2

**Published:** 2016-04-12

**Authors:** N. Dasgupta-Schubert, D. K. Tiwari, L. M. Villaseñor Cendejas

**Affiliations:** Instituto de Investigaciones Químico Biológicas (IIQB), University of Michoacan (UMSNH), Cd. Universitaria, 58060 Morelia, Michoacán Mexico; Colegio de Michoacán, Jardines del Cerro Grande, La Piedad, 59370 Zamora, Michoacán Mexico; Instituto de Física y Matemáticas (IFM), University of Michoacan (UMSNH), Cd. Universitaria, 58060 Morelia, Michoacán Mexico

**Keywords:** Carbon nanotubes, Fe(II), Fe(III), Iron oxidation

## Abstract

A recent review article entitled “Carbon and fullerene nanomaterials in plant system” published in this journal, misinterprets a component of our (published) work on the interactions of carbon nanotubes with plants. In this comment, we provide the rationale to counter this misconstruction.

## Background

In page seven of their review article [1] Azamal Husen and Salahuddin Sidiqi say the following in the context of our work [2]: “The authors have shown changes in the morphology of MWCNTs after the addition of Fe^2+^ and Fe^3+^ in separate experiments. Since Fe^2+^ in aqueous medium is immediately oxidized to Fe^3+^, the effect of addition of Fe^2+^ will exactly be the same as that of Fe^3+^. However, Fe^2+^ can be stabilized in acidic medium.” While the rapid oxidation of Fe^2+^ to Fe^3+^ is certainly true in aqueous alkaline media, their statement in the context of the particulars of our work is incorrect. To wit, in our work the Fe^2+^ was not *immediately* oxidized to Fe^3+^ as we proceed to explain below. Consequently, the observations regarding the influence of the oxidation state of dissolved iron on the physiological response of the germinating seedling as detailed in [2] stand substantiated. Moreover, there is an obvious slip in their first sentence. The phrase “morphology of the MWCNT” should be replaced by “morphology of the MWCNT *treated seedlings*”. The letters MWCNT stand for multi-walled carbon nanotubes.

The oxidation of Fe^2+^ to Fe^3+^ (henceforth designated as Fe(II) and Fe(III)) has been extensively studied [3–5]. The general idea about the kinetics that emerges is that the oxidation of Fe(II) (aq) is first order with respect to the Fe(II) concentration and the O_2_ partial pressure (p_O2_), while it is second order with respect to the OH^−^ concentration, in the pH range of 5.5–7.5. For more acidic or alkaline pHs, the rate of oxidation is nearly independent of pH.

1 $$- {\text{d}}\left[ {{\text{Fe}}\left( {\text{II}} \right)} \right]/{\text{dt}} = {\text{k }}\left[ {{\text{Fe}}\left( {\text{II}} \right)} \right].{\text{ p}}_{\text{O2}} \left[ {{\text{OH}}^{ - } } \right]^{ 2}$$

For a constant pH and in the presence of excess oxygen, Eq. (1) reduces to a pseudo first order equation,

2 $$- {\text{d}}\left[ {{\text{Fe}}\left( {\text{II}} \right)} \right]/{\text{dt}} = {\text{k}}_{0} \left[ {{\text{Fe}}\left( {\text{II}} \right)} \right]$$

where the rate constant is,

3 $${\text{k}}_{\text{o}} = - {\text{d}}\left( {{ \ln }\left[ {{\text{Fe}}\left( {\text{II}} \right)} \right]} \right)/{\text{dt}} = - 2. 30 3 {\text{ d}}\left( {{ \log }\left[ {{\text{Fe}}\left( {\text{II}} \right)} \right]} \right)/{\text{dt}} = 2. 30 3 {\text{k}}''$$

In our work [2] the pH of the DI water used to constitute the agarose medium, was 6.3. After the addition of the iron(II) chloride the agarose solution would have become slightly further acidic on account of the iron (II) chloride being a weak Lewis acid [6]. However for the purpose of the present analysis the pH will be considered to be ~ 6.3. The normal atmospheric partial pressure of O_2_ is 0.209 atm and O_2_ is the reagent in excess. Hence we use the first order rate constant k″.

While details of the experiment are described in [2], a few points salient to the present discussion, bear mentioning.

The agarose solution was allowed to cool to about 40 °C (313.15 K) which was above the gelling temperature. A mass of fresh iron(II) chloride (Sigma Aldrich) appropriate for a concentration of 3.0 × 10^−4^ M in the medium, and the appropriate mass of MWCNT, were added with constant stirring until the complete dissolution of the iron(II) chloride (duration of step ~ 10 min). The solution was then poured out into the appropriate petridishes and allowed to cool to room temperature ~ 25 °C (298.15 K) whereupon it formed the iron chloride and MWCNT doped gel (duration ~ 15 min). The surface sterilized seeds were then embedded in the gel (duration ~ 10 min). The net time the iron (II) chloride was exposed to oxidation in the agarose medium was therefore ~ 35 min.

Thereafter the Fe(II) would enter the seed coat by diffusion which would take the time t_D_ depending on the thickness of the seed-coat, x_SC_, and the coefficient of diffusion of the Fe(II), D_Fe_. The thickness of the seed-coats of seeds can vary from several microns to some fractions of a mm. We assume a maximum value of x_SC_ as 1 mm so as to set an upper limit on t_D_. Most dipositive metal cations have an aqueous phase diffusion coefficient of the order of 10^−9^ m^2^s^−1^. The orifices of the seed-coat through which the Fe(II) would enter the physiologically active parts of the seed are largely filled with water so following Nobel [7], it suffices to use the diffusion coefficient for aqueous media. We assume a value of D_Fe_ as identical to the value for Ca^2+^ which is 1.2 × 10^−9^ m^2^ s^−1^ [7]. Using these values and the equation for (planar) diffusion [7],

4 $${\text{x}}_{\text{SC}}^{ 2} = 4.{\text{ D}}_{\text{Fe}} .{\text{ t}}_{\text{D}}$$

we obtain t_D_ as ~ 4 min. Hence the total time that the Fe(II) is available for oxidation in the aqueous medium is ~ 39 min. Figure 1 in [5] pertains to the variation of log k″ with pH at T = 298.15 K and a p_O2_ of 0.20 atm. These conditions apply to our experiment. Reading off their data at pH ~ 6.3, we obtain k″ (298.15 K) as 2.196 × 10^−3^ min^−1^. Using this value and integrating the differential in Eq. 3 where the concentration limits are the logs of the initial and final concentrations of Fe(II) (the initial concentration being 3.0 × 10^−4^ M), and the corresponding time limits 0 and 39 min., we get the final concentration of Fe(II) after it has suffered oxidation, as 2.46 × 10^−4^ M.

However for its duration of stay in the agarose medium, the temperature endured by the iron (II) chloride was greater than 25 °C. We will assume an upper limit of 313.15 K throughout its stay as this will give us the maximum possible reduction in the Fe(II) concentration by oxidation. However, once the agarose had gelled and the Fe began diffusing into the seed-coat, the temperature was 298.15 K. Trapp and Millero [4] present data for the oxidation of nanomolar concentrations of Fe(II) in the presence of brine solution. They show that the variation of the log of the pseudo first order rate constant of oxidation is linear with respect to 1/T throughout the range of concentrations of Fe(II) and brine at constant pH and excess concentrations of gaseous oxygen. Using their temperature data for logk″ for the pH = 6.3, we obtain,

5 $${\text{logk}}{''} = - 1 7 8 9 { }\left( { 1/{\text{T}}} \right) + 3. 6 7 7$$

For dilute Fe(II) solutions (as in our work) under the same conditions of excess oxygen and in the same limited temperature range, the above equation is expected to hold with differences, if any, arising only in the constant term for non-reactants (such as brine). Hence for use in our work Eq. 5 was re-written as follows, avoiding the constant term:

6 $${\text{logk}}{''}\left( {{\text{T}}_{ 2} } \right){-}{\text{logk}}{''}\left( {{\text{T}}_{ 1} } \right) = - 1 7 8 9\{ \left( { 1/{\text{T}}_{ 2} } \right){-}\left( { 1/{\text{T}}_{ 1} } \right)\}$$

With T_1_, T_2_, k″(T_1_) being 298.15 K, 313.15 K and 2.196 × 10^−3^ min^−1^ respectively, k″(313.15 K) becomes 4.263 × 10^−3^ min^−1^. We used this value to calculate the upper limit of the reduction in the Fe(II) concentration by oxidation in the agarose medium in the first 35 min. The further reduction as a consequence of the 4 min of diffusion across the seed-coat was calculated as before at the temperature of 25 °C (see above). Thus overall, the Fe(II) concentration reduced to 2.085 × 10^−4^ M.

Thus we see that even assuming exaggeratedly adverse conditions (constant high temperature in the agarose medium, high seed-coat thickness, a pH more alkaline than the actual), the Fe(II) retains − ~ 70 % of its initial concentration. It did not *immediately* oxidise to Fe(III). The aforesaid result signifies that despite undergoing some oxidation to Fe(III), the Fe(II) was still present at a preponderant level at the moment when it presented itself for interaction with the physiologically relevant biomolecules within the seed. This would influence later physiological developments. Hence the experimental observations of a *difference* in response between maize seeds treated with MWCNT and iron (II) and iron (III) chlorides as described in [2] are valid.

## Availability of data and materials

The description of the data cited in this work, is available in the public-domain publications given in the Reference section of this manuscript. Further details on the experimental work that forms the central topic of this manuscript, are to be found in the lines 52-60.

## Abbreviation

MWCNT: multi-walled carbon nanotubes
